# {2,6-Bis[(pyridin-2-yl)sulfanylmeth­yl]pyridine-κ^2^ *N*,*N*′}(η^3^-prop-2-en­yl)palladium(II) hexa­fluoro­phosphate

**DOI:** 10.1107/S1600536814003961

**Published:** 2014-03-15

**Authors:** Giuseppe Bruno, Francesco Nicolò, Giuseppe Tresoldi, Dario Drommi, Viviana Mollica Nardo

**Affiliations:** a Dipartimento di Scienze Chimiche, Universita di Messina, Messina, Italy

## Abstract

The title compound, [Pd(C_3_H_5_)(C_17_H_15_N_3_S_2_)]PF_6_, is built up by a [(η^3^-all­yl)Pd]^2+^ fragment coordinated by a 2,6-bis­[(pyridin-2-yl)sulfanylmeth­yl]pyridine ligand coordinated through the N atoms. One of the S atoms is at a close distance to the metal centeratom [3.2930 (8) Å]. The Pd^II^ atom is tetra­coordinated in a strongly distorted square-planar environment mainly determined by the η^3^-allyl anion in which the central C atom is disordered over two equally occupied positions. The crystal packing is very compact and is characterized by a three-dimensional network of C—H⋯F interactions between the F atoms of each anion and several H atoms of the surrounding cationic complexes.

## Related literature   

For the catalitic use of palladium η^3^-allyl complexes, see: De Vries (2012 ▶). For multidentate nitro­gen/sulfur ligands, see: Betz *et al.* (2008 ▶). For the η^3^–η^1^–η^3^ mechanism and *syn-*-*syn* anti–anti isomerism, see: Solin & Szabo (2001 ▶); Takao *et al.* (2003 ▶); Barloy *et al.* (2000 ▶); Gogoll *et al.* (1997 ▶); Tresoldi *et al.* (2008 ▶, 2010 ▶). For the synthesis and structural properties of palladium–allyl complexes, see: Scopelliti *et al.* (2001 ▶); Tresoldi *et al.* (2002 ▶); Baradello *et al.* (2004 ▶).
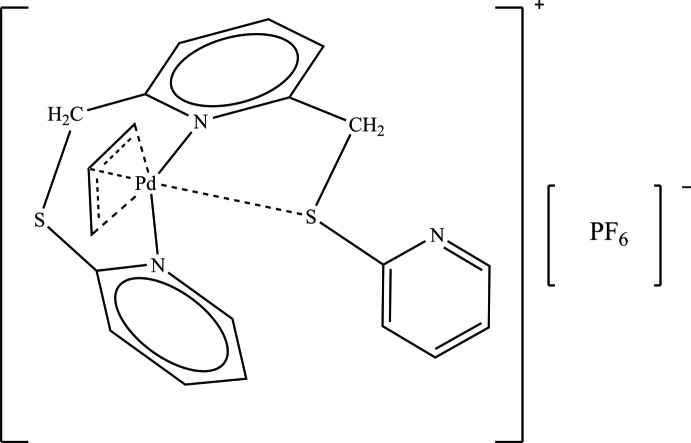


## Experimental   

### 

#### Crystal data  

[Pd(C_3_H_5_)(C_17_H_15_N_3_S_2_)]PF_6_
*M* *_r_* = 617.88
Monoclinic, 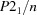

*a* = 13.663 (1) Å
*b* = 13.667 (1) Å
*c* = 13.727 (1) Å
β = 113.01 (1)°
*V* = 2359.3 (3) Å^3^
*Z* = 4
Mo *K*α radiation
μ = 1.09 mm^−1^
*T* = 293 K
0.4 × 0.32 × 0.29 mm

#### Data collection  

Bruker APEXII CCD diffractometer
Absorption correction: integration (*SADABS*; Bruker, 2005 ▶) *T*_min_ = 0.634, *T*_max_ = 0.666
74899 measured reflections
5419 independent reflections
4912 reflections with *I* > 2σ(*I*)
*R*_int_ = 0.028

#### Refinement  

*R*[*F* ^2^ > 2σ(*F* ^2^)] = 0.038
*wR*(*F* ^2^) = 0.114
*S* = 1.07
5419 reflections
307 parameters
H-atom parameters constrained
Δρ_max_ = 0.57 e Å^−3^
Δρ_min_ = −0.69 e Å^−3^

### 

Data collection: *APEX2* (Bruker, 2005 ▶); cell refinement: *SAINT* (Bruker, 2005 ▶); data reduction: *SAINT*; program(s) used to solve structure: *SHELXS97* (Sheldrick, 2008 ▶); program(s) used to refine structure: *SHELXL97* (Sheldrick, 2008 ▶); molecular graphics: *SHELXTL* (Sheldrick, 2008 ▶); software used to prepare material for publication: *SHELXTL*.

## Supplementary Material

Crystal structure: contains datablock(s) I, New_Global_Publ_Block. DOI: 10.1107/S1600536814003961/bt6949sup1.cif

Structure factors: contains datablock(s) I. DOI: 10.1107/S1600536814003961/bt6949Isup2.hkl

CCDC reference: 988035

Additional supporting information:  crystallographic information; 3D view; checkCIF report

## Figures and Tables

**Table 1:** Hydrogen-bond geometry (Å, °)

*D*—H⋯*A*	*D*—H	H⋯*A*	*D*⋯*A*	*D*—H⋯*A*
C3—H3⋯F3^i^	0.93	2.42	3.270 (8)	151
C4—H4⋯F2^ii^	0.93	2.47	3.358 (6)	161
C8—H8⋯F4^iii^	0.93	2.44	3.317 (8)	157
C12—H12*A*⋯F3^iv^	0.97	2.45	3.126 (8)	126

Symmetry codes: (i) 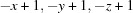; (ii) 
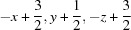
; (iii) 
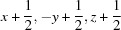
; (iv) 
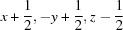
.

**Fig. 1.**
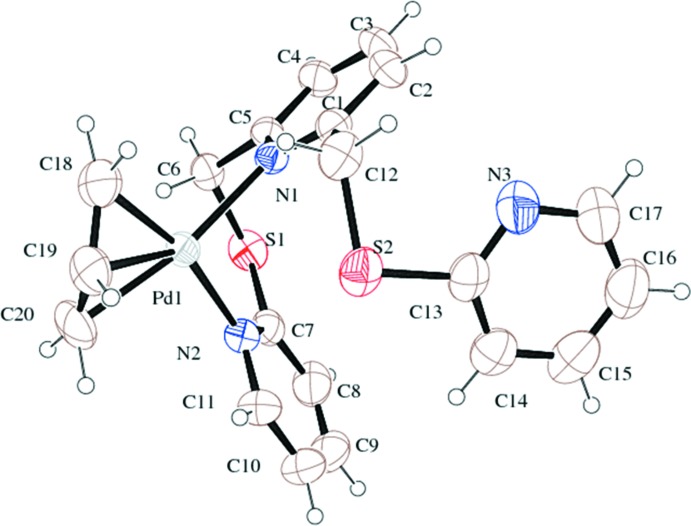
Perspective view of the title molecule with numbering of the atoms. Non H-atoms represented as displacement ellipsoids are plotted at the 30% probability level, while H atoms are shown as small spheres of arbitrary radius. The PF6- anion and one of the disordered C atoms have been omitted for clarity.

## supplementary crystallographic information

### 1. Comment

The chemistry of palladium-allyl complexes has been reported extensively. This interest has stemmed from the relevance to palladium-catalyzed allylic substitution reactions. Attack of the nucleophile on a cationic palladium η^3^-allyl complex is conventionally accepted as the crucial step in the catalytic cycle (De Vries 2012). Multidentate nitrogen ligands or multidentate sulfur nitrogen ligands have been used for the structure analysis of (η^3^-allyl)palladium complexes and the study of their influence upon the dynamic behaviour (Betz *et al.*, 2008). One process encountered in (η^3^-allyl)palladium complexes is a *syn* anti isomerism which has been subject of research for many years. The η^3^-η^1^-η^3^ mechanism is well established for many [Pd(η^3^-allyl)*L*1*L*2]^+^ cations (Solin *et al.*, 2001). Other process usually observed is apparent allyl rotation or *syn*-*syn* anti-anti isomerism (Takao *et al.*, 2003). Mechanisms proposed for this process include: i) an associative mechanism, involving a non-rigid five-coordinated intermediate (Barloy *et al.*, 2000), ii) a dissociative mechanism with stabilization of tricoordinate intermediate, mostly, in some complexes with N-donor ligands (Gogoll *et al.*, 1997). We have lately been interested in the use of potentially tridentate N, S, N ligands in coordination chemistry towards congested ruthenium(II)substrates. The contemporary presence of the congested substrate and sterically demanding ligand favoured the *N*,*S*-chelation of the thioether with formation of the four membered chelate ring (Scopelliti *et al.*, 2001; Tresoldi *et al.*, 2002) and the five membered chelate ring, (Baradello *et al.*, 2004). However the exchange between these isomers was hampered by the very low abundance of the four membered species and the large value of the activation energy of the process (Tresoldi *et al.*, 2008). The potentially polidentate N_3_S_2_ or N_5_S_2_ dithioethers, containing a 2,6-substitute linker with two thioether-heterocycle arms, were prepared and their reactions with some congested ruthenium substrates explored. Only the four membered species was obtained and the sulfur inversion observed. (Tresoldi *et al.*, 2010) With the aim of favouring different modes of coordination of dithioethers and as extension of our stereodynamic studies, we have examined the reactions of the dithioethers with the solvate species [Pd(allyl)(acetone)_2_]^+^, obtaining the species [Pd(allyl)(dithioether)]PF_6_ stereo-chemically no-rigid. The Nouter, Ncentral-coordination of dithioether ligand with formation of seven membered chelate ring is favoured in the solid state while in solution the dynamic behaviour of the compounds involves the dithioether attempt to coordinate with all the donor atoms. In this paper the reaction of 2,6-bis(2-pyridylsulfanylmethyl)pyridine(psmp) with [Pd(C_3_H_5_)(acetone)]^+^, generate *in situ* from [Pd(C_3_H_5_)Cl]_2_ and AgPF_6_, led to the title compound,[Pd(C_3_H_5_)(psmp)]PF_6_ (I).

### 2. Experimental

To a stirred solution, of [Pd(C_3_H_5_)Cl]_2_ (91,5 mg, 0.25 mmol) in acetone (20 ml) was added AgPF_6_ (126.4 mg, 0.5 mmol). After 30 m silver chloride was filtered. The solution of the solvento species was added to 15 ml of acetone containing 162.7 mg (0.5 mmol) of psmp and stirred for 2 h. The resulting solution was filterd on diethyl ether (120 ml) and a white precipitate was obtained, which was filtered, washed with diethyl ether (30 ml) and dried overnight. Yield 247 mg (80%). White crystals of I suitable for X-ray structure investigation were obtained from a solution in acetone-ether (1:3) on standing for *ca* 4 d at 253 K°. C_20_H_20_F_6_N_3_PPdS_2_ (617.9) requires: C 38.88, H 3.26, N 6.80, S 10.38%; found: C 38.80, H 3.30, N 6.90, S 10.70%. Conductivity: ΛM(MeCN, 5x10–4 mol *L*-1, 20 °C) = 146 S *cm*2 mol-1. 1H NMR (500 MHz, [D6]acetone, 323 °C): δ8.65 (dd, J6,4 = 1.6 Hz, J_6,5_ = 5.4 Hz, 2H, H6),8.02 (t, J = 7.8 Hz, 1H, H4'), 7.76 (d, 2H, H3', H5'), 7.67 (ddd, J4,5 = 7.8 Hz, J_4,3_ = 8.2 Hz, 2H, H4), 7.38 (ddd, J_5,3_ = 1.0 Hz, 2H, H3), 7.19 (ddd, 2H, H5), 6.13 (m, 1H, allyl CH), 5.14 (s, 4H, S—CH2), 3.97 (br, 4H, allyl *syn*-anti).

### 3. Refinement

H atoms were included in the refinement using a riding model method with the *X*—H bond geometry and the H isotropic displacement parameter depending on the parent atom *X*. One C atom of the allyl moiety is disordered over two equally occupied positions.

### Crystal data

**Table:**

[Pd(C_3_H_5_)(C_17_H_15_N_3_S_2_)]PF_6_	*F*(000) = 1232
*M**_r_* = 617.88	*D*_x_ = 1.739 Mg m^−^^3^
Monoclinic, *P*2_1_/*n*	Mo *K*α radiation, λ = 0.71073 Å
Hall symbol: -P 2yn	Cell parameters from 136 reflections
*a* = 13.663 (1) Å	θ = 2.2–30.0°
*b* = 13.667 (1) Å	µ = 1.09 mm^−^^1^
*c* = 13.727 (1) Å	*T* = 293 K
β = 113.01 (1)°	Prismatic, yellow
*V* = 2359.3 (3) Å^3^	0.4 × 0.32 × 0.29 mm
*Z* = 4	

### Data collection

**Table:**

Bruker APEXII CCD diffractometer	5419 independent reflections
Radiation source: fine-focus sealed tube	4912 reflections with *I* > 2σ(*I*)
Graphite monochromator	*R*_int_ = 0.028
φ and ω scans	θ_max_ = 27.5°, θ_min_ = 2.2°
Absorption correction: integration (*SADABS*; Bruker, 2005)	*h* = −17→17
*T*_min_ = 0.634, *T*_max_ = 0.666	*k* = −17→17
74899 measured reflections	*l* = −17→17

### Refinement

**Table:**

Refinement on *F*^2^	Primary atom site location: structure-invariant direct methods
Least-squares matrix: full	Secondary atom site location: difference Fourier map
*R*[*F*^2^ > 2σ(*F*^2^)] = 0.038	Hydrogen site location: inferred from neighbouring sites
*wR*(*F*^2^) = 0.114	H-atom parameters constrained
*S* = 1.07	*w* = 1/[σ^2^(*F*_o_^2^) + (0.0579*P*)^2^ + 2.4402*P*] where *P* = (*F*_o_^2^ + 2*F*_c_^2^)/3
5419 reflections	(Δ/σ)_max_ = 0.002
307 parameters	Δρ_max_ = 0.57 e Å^−^^3^
0 restraints	Δρ_min_ = −0.69 e Å^−^^3^

### Special details

**Table:**

Geometry. All esds (except the esd in the dihedral angle between two l.s. planes) are estimated using the full covariance matrix. The cell esds are taken into account individually in the estimation of esds in distances, angles and torsion angles; correlations between esds in cell parameters are only used when they are defined by crystal symmetry. An approximate (isotropic) treatment of cell esds is used for estimating esds involving l.s. planes.
Refinement. Refinement of *F*^2^ against ALL reflections. The weighted *R*-factor *wR* and goodness of fit *S* are based on *F*^2^, conventional *R*-factors *R* are based on *F*, with *F* set to zero for negative *F*^2^. The threshold expression of *F*^2^ > 2σ(*F*^2^) is used only for calculating *R*-factors(gt) *etc*. and is not relevant to the choice of reflections for refinement. *R*-factors based on *F*^2^ are statistically about twice as large as those based on *F*, and *R*- factors based on ALL data will be even larger.

### Fractional atomic coordinates and isotropic or equivalent isotropic displacement parameters (Å^2^)

**Table:**

	*x*	*y*	*z*	*U*_iso_*/*U*_eq_	Occ. (<1)
Pd1	0.88269 (2)	0.24170 (2)	0.51700 (2)	0.04910 (10)	
S1	0.92247 (8)	0.38395 (7)	0.76617 (7)	0.0673 (2)	
S2	0.63967 (7)	0.29098 (8)	0.34786 (9)	0.0729 (3)	
P1	0.33006 (8)	0.11910 (9)	0.58509 (8)	0.0708 (3)	
N2	0.8081 (2)	0.23926 (19)	0.6281 (3)	0.0568 (6)	
N1	0.85839 (18)	0.39548 (17)	0.49428 (18)	0.0434 (5)	
C6	0.9869 (2)	0.4005 (2)	0.6749 (2)	0.0540 (7)	
H6A	1.0516	0.4381	0.7090	0.065*	
H6B	1.0064	0.3372	0.6561	0.065*	
C5	0.9163 (2)	0.4522 (2)	0.5770 (2)	0.0472 (6)	
C1	0.7881 (2)	0.4391 (2)	0.4067 (2)	0.0520 (6)	
C7	0.8199 (3)	0.2976 (3)	0.7103 (3)	0.0589 (8)	
C2	0.7764 (3)	0.5391 (3)	0.4006 (3)	0.0678 (9)	
H2	0.7273	0.5677	0.3396	0.081*	
C3	0.8370 (3)	0.5967 (3)	0.4841 (4)	0.0737 (10)	
H3	0.8303	0.6645	0.4801	0.088*	
C12	0.7244 (3)	0.3743 (3)	0.3162 (3)	0.0686 (9)	
H12A	0.7726	0.3371	0.2941	0.082*	
H12B	0.6810	0.4148	0.2570	0.082*	
C14	0.4859 (3)	0.3416 (4)	0.4160 (3)	0.0794 (11)	
H14	0.5013	0.2825	0.4524	0.095*	
C13	0.5421 (3)	0.3717 (3)	0.3584 (3)	0.0688 (9)	
C4	0.9077 (3)	0.5525 (2)	0.5735 (3)	0.0626 (8)	
H4	0.9495	0.5900	0.6313	0.075*	
C15	0.4064 (4)	0.4005 (5)	0.4192 (5)	0.1015 (16)	
H15	0.3664	0.3821	0.4576	0.122*	
C11	0.7291 (3)	0.1721 (3)	0.6012 (4)	0.0767 (10)	
H11	0.7209	0.1299	0.5454	0.092*	
C9	0.6727 (4)	0.2240 (5)	0.7335 (5)	0.0987 (17)	
H9	0.6269	0.2192	0.7687	0.118*	
N3	0.5268 (3)	0.4554 (3)	0.3068 (3)	0.0924 (11)	
C8	0.7514 (4)	0.2918 (4)	0.7639 (4)	0.0842 (12)	
H8	0.7597	0.3341	0.8197	0.101*	
C10	0.6609 (4)	0.1628 (4)	0.6513 (5)	0.0942 (15)	
H10	0.6076	0.1157	0.6298	0.113*	
C17	0.4487 (5)	0.5119 (5)	0.3121 (6)	0.118 (2)	
H17	0.4362	0.5720	0.2776	0.141*	
C16	0.3869 (4)	0.4866 (5)	0.3648 (6)	0.1136 (19)	
H16	0.3321	0.5274	0.3640	0.136*	
F1	0.2921 (4)	0.0312 (3)	0.6341 (3)	0.1496 (15)	
F6	0.3721 (4)	0.2025 (4)	0.5351 (4)	0.175 (2)	
F2	0.4174 (3)	0.1344 (4)	0.6926 (3)	0.1620 (18)	
F3	0.2562 (5)	0.1880 (5)	0.6114 (5)	0.225 (3)	
F5	0.3954 (5)	0.0555 (5)	0.5459 (5)	0.218 (3)	
F4	0.2372 (4)	0.1053 (6)	0.4768 (4)	0.248 (4)	
C18	0.9545 (4)	0.2038 (3)	0.4089 (4)	0.0780 (11)	
H18A	1.0023	0.2399	0.4647	0.094*	0.5
H18B	0.9384	0.2234	0.3395	0.094*	0.5
H18C	0.8832	0.2196	0.3725	0.094*	0.5
H18D	1.0060	0.2322	0.3894	0.094*	0.5
C19	0.9081 (6)	0.1225 (5)	0.4289 (6)	0.0578 (16)	0.5
H19	0.8603	0.0866	0.3729	0.069*	0.5
C19A	0.9822 (11)	0.1423 (7)	0.4872 (13)	0.111 (4)	0.5
H19A	1.0552	0.1318	0.5180	0.133*	0.5
C20	0.9312 (4)	0.0929 (3)	0.5311 (4)	0.0826 (12)	
H20A	0.9789	0.1286	0.5873	0.099*	0.5
H20B	0.8991	0.0372	0.5441	0.099*	0.5
H20C	0.8575	0.0980	0.5064	0.099*	0.5
H20D	0.9676	0.0519	0.5878	0.099*	0.5

### Atomic displacement parameters (Å^2^)

**Table:**

	*U*^11^	*U*^22^	*U*^33^	*U*^12^	*U*^13^	*U*^23^
Pd1	0.05044 (15)	0.03748 (13)	0.06055 (17)	−0.00211 (8)	0.02296 (11)	−0.00752 (8)
S1	0.0830 (6)	0.0675 (5)	0.0497 (4)	0.0056 (4)	0.0242 (4)	−0.0034 (4)
S2	0.0549 (5)	0.0693 (6)	0.0831 (6)	−0.0076 (4)	0.0145 (4)	−0.0209 (5)
P1	0.0635 (5)	0.0844 (7)	0.0655 (5)	−0.0011 (5)	0.0264 (4)	0.0102 (5)
N2	0.0555 (15)	0.0507 (14)	0.0676 (17)	0.0019 (11)	0.0279 (13)	0.0082 (12)
N1	0.0420 (11)	0.0404 (11)	0.0485 (11)	−0.0026 (9)	0.0184 (9)	−0.0029 (9)
C6	0.0487 (15)	0.0502 (16)	0.0546 (16)	−0.0003 (12)	0.0110 (12)	−0.0052 (13)
C5	0.0454 (13)	0.0422 (14)	0.0543 (15)	−0.0037 (11)	0.0198 (12)	−0.0038 (11)
C1	0.0463 (14)	0.0601 (17)	0.0497 (14)	−0.0007 (12)	0.0190 (12)	0.0035 (13)
C7	0.0612 (18)	0.0620 (19)	0.0571 (17)	0.0161 (15)	0.0271 (14)	0.0180 (15)
C2	0.0612 (19)	0.064 (2)	0.073 (2)	0.0070 (16)	0.0207 (17)	0.0240 (17)
C3	0.075 (2)	0.0418 (16)	0.101 (3)	0.0021 (16)	0.030 (2)	0.0125 (17)
C12	0.0597 (19)	0.089 (3)	0.0497 (17)	0.0001 (18)	0.0132 (14)	−0.0067 (17)
C14	0.071 (2)	0.080 (3)	0.082 (3)	−0.011 (2)	0.025 (2)	−0.018 (2)
C13	0.0490 (16)	0.074 (2)	0.068 (2)	−0.0065 (16)	0.0065 (15)	−0.0186 (18)
C4	0.0656 (19)	0.0416 (15)	0.078 (2)	−0.0077 (14)	0.0252 (17)	−0.0061 (15)
C15	0.080 (3)	0.113 (4)	0.121 (4)	−0.014 (3)	0.049 (3)	−0.031 (3)
C11	0.072 (2)	0.064 (2)	0.096 (3)	−0.0087 (18)	0.035 (2)	0.010 (2)
C9	0.082 (3)	0.123 (4)	0.114 (4)	0.019 (3)	0.063 (3)	0.048 (4)
N3	0.074 (2)	0.093 (3)	0.100 (3)	0.015 (2)	0.0233 (19)	0.010 (2)
C8	0.087 (3)	0.106 (3)	0.072 (2)	0.027 (3)	0.045 (2)	0.026 (2)
C10	0.072 (3)	0.094 (3)	0.126 (4)	−0.003 (2)	0.048 (3)	0.036 (3)
C17	0.089 (3)	0.102 (4)	0.153 (5)	0.028 (3)	0.039 (4)	0.022 (4)
C16	0.079 (3)	0.112 (5)	0.147 (5)	0.019 (3)	0.041 (3)	−0.012 (4)
F1	0.173 (4)	0.129 (3)	0.151 (3)	−0.037 (3)	0.068 (3)	0.034 (2)
F6	0.203 (5)	0.191 (5)	0.145 (3)	−0.071 (4)	0.083 (3)	0.039 (3)
F2	0.135 (3)	0.210 (5)	0.094 (2)	−0.044 (3)	−0.006 (2)	0.003 (3)
F3	0.278 (6)	0.211 (5)	0.264 (6)	0.164 (5)	0.192 (6)	0.149 (5)
F5	0.234 (6)	0.219 (6)	0.264 (6)	0.033 (5)	0.167 (5)	−0.062 (5)
F4	0.142 (4)	0.448 (11)	0.115 (3)	−0.104 (5)	0.009 (3)	0.073 (5)
C18	0.089 (3)	0.069 (2)	0.092 (3)	0.005 (2)	0.053 (2)	−0.017 (2)
C19	0.056 (4)	0.047 (3)	0.070 (4)	−0.001 (3)	0.024 (3)	−0.025 (3)
C19A	0.134 (10)	0.057 (5)	0.189 (13)	0.028 (6)	0.115 (10)	0.002 (7)
C20	0.106 (3)	0.0407 (17)	0.105 (3)	0.0147 (19)	0.046 (3)	−0.0080 (18)

### Geometric parameters (Å, º)

**Table:**

Pd1—C19A	2.073 (8)	C14—C13	1.364 (6)
Pd1—C20	2.124 (4)	C14—C15	1.365 (7)
Pd1—N1	2.132 (2)	C14—H14	0.9300
Pd1—C18	2.137 (4)	C13—N3	1.318 (6)
Pd1—C19	2.136 (6)	C4—H4	0.9300
Pd1—N2	2.141 (3)	C15—C16	1.364 (8)
S1—C7	1.761 (4)	C15—H15	0.9300
S1—C6	1.805 (3)	C11—C10	1.363 (6)
S2—C13	1.779 (4)	C11—H11	0.9300
S2—C12	1.795 (4)	C9—C8	1.357 (8)
P1—F5	1.489 (4)	C9—C10	1.363 (8)
P1—F2	1.506 (3)	C9—H9	0.9300
P1—F3	1.523 (5)	N3—C17	1.344 (7)
P1—F4	1.543 (4)	C8—H8	0.9300
P1—F6	1.553 (4)	C10—H10	0.9300
P1—F1	1.561 (4)	C17—C16	1.353 (8)
N2—C7	1.339 (5)	C17—H17	0.9300
N2—C11	1.353 (5)	C16—H16	0.9300
N1—C5	1.347 (4)	C18—C19A	1.299 (14)
N1—C1	1.350 (4)	C18—C19	1.360 (9)
C6—C5	1.490 (4)	C18—H18A	0.9300
C6—H6A	0.9700	C18—H18B	0.9300
C6—H6B	0.9700	C18—H18C	0.9300
C5—C4	1.375 (4)	C18—H18D	0.9300
C1—C2	1.374 (5)	C19—C20	1.373 (9)
C1—C12	1.497 (5)	C19—H19	0.9300
C7—C8	1.401 (5)	C19A—C20	1.278 (12)
C2—C3	1.370 (6)	C19A—H19A	0.9300
C2—H2	0.9300	C20—H20A	0.9300
C3—C4	1.370 (5)	C20—H20B	0.9300
C3—H3	0.9300	C20—H20C	0.9300
C12—H12A	0.9700	C20—H20D	0.9300
C12—H12B	0.9700		
			
C19A—Pd1—C20	35.4 (3)	C15—C14—H14	120.8
C19A—Pd1—N1	134.0 (3)	N3—C13—C14	124.5 (4)
C20—Pd1—N1	169.28 (15)	N3—C13—S2	117.4 (3)
C19A—Pd1—C18	35.9 (4)	C14—C13—S2	118.1 (4)
C20—Pd1—C18	67.68 (19)	C3—C4—C5	119.5 (3)
N1—Pd1—C18	102.98 (14)	C3—C4—H4	120.3
C20—Pd1—C19	37.6 (2)	C5—C4—H4	120.3
N1—Pd1—C19	136.0 (2)	C16—C15—C14	118.6 (5)
C18—Pd1—C19	37.1 (2)	C16—C15—H15	120.7
C19A—Pd1—N2	131.5 (4)	C14—C15—H15	120.7
C20—Pd1—N2	97.96 (15)	N2—C11—C10	123.7 (5)
N1—Pd1—N2	91.68 (9)	N2—C11—H11	118.2
C18—Pd1—N2	165.10 (15)	C10—C11—H11	118.2
C19—Pd1—N2	128.3 (2)	C8—C9—C10	119.8 (4)
C7—S1—C6	107.62 (15)	C8—C9—H9	120.1
C13—S2—C12	101.6 (2)	C10—C9—H9	120.1
F5—P1—F2	94.4 (3)	C13—N3—C17	115.6 (5)
F5—P1—F3	173.1 (4)	C9—C8—C7	119.3 (5)
F2—P1—F3	91.5 (4)	C9—C8—H8	120.3
F5—P1—F4	88.0 (4)	C7—C8—H8	120.3
F2—P1—F4	177.5 (4)	C9—C10—C11	118.6 (5)
F3—P1—F4	86.1 (4)	C9—C10—H10	120.7
F5—P1—F6	83.8 (4)	C11—C10—H10	120.7
F2—P1—F6	92.4 (3)	N3—C17—C16	123.8 (6)
F3—P1—F6	92.6 (3)	N3—C17—H17	118.1
F4—P1—F6	88.3 (3)	C16—C17—H17	118.1
F5—P1—F1	93.0 (3)	C17—C16—C15	119.0 (5)
F2—P1—F1	87.4 (2)	C17—C16—H16	120.5
F3—P1—F1	90.7 (3)	C15—C16—H16	120.5
F4—P1—F1	92.0 (3)	C19A—C18—Pd1	69.3 (4)
F6—P1—F1	176.8 (3)	C19—C18—Pd1	71.4 (3)
C7—N2—C11	117.1 (3)	C19—C18—H18A	120.0
C7—N2—Pd1	131.0 (2)	Pd1—C18—H18A	70.5
C11—N2—Pd1	111.5 (3)	C19—C18—H18B	120.0
C5—N1—C1	118.4 (3)	Pd1—C18—H18B	130.8
C5—N1—Pd1	115.92 (19)	H18A—C18—H18B	120.0
C1—N1—Pd1	125.6 (2)	C19A—C18—H18C	120.0
C5—C6—S1	111.5 (2)	Pd1—C18—H18C	69.5
C5—C6—H6A	109.3	C19A—C18—H18D	120.0
S1—C6—H6A	109.3	Pd1—C18—H18D	134.7
C5—C6—H6B	109.3	H18C—C18—H18D	120.0
S1—C6—H6B	109.3	C18—C19—C20	120.5 (6)
H6A—C6—H6B	108.0	C18—C19—Pd1	71.5 (3)
N1—C5—C4	122.0 (3)	C20—C19—Pd1	70.7 (3)
N1—C5—C6	116.6 (3)	C18—C19—H19	119.7
C4—C5—C6	121.4 (3)	C20—C19—H19	119.7
N1—C1—C2	121.2 (3)	Pd1—C19—H19	130.8
N1—C1—C12	117.3 (3)	C20—C19A—C18	134.1 (12)
C2—C1—C12	121.5 (3)	C20—C19A—Pd1	74.5 (4)
N2—C7—C8	121.5 (4)	C18—C19A—Pd1	74.7 (5)
N2—C7—S1	125.4 (3)	C20—C19A—H19A	113.0
C8—C7—S1	113.0 (3)	C18—C19A—H19A	113.0
C3—C2—C1	120.2 (3)	Pd1—C19A—H19A	132.9
C3—C2—H2	119.9	C19A—C20—Pd1	70.1 (4)
C1—C2—H2	119.9	C19—C20—Pd1	71.7 (3)
C4—C3—C2	118.7 (3)	C19—C20—H20A	120.0
C4—C3—H3	120.7	Pd1—C20—H20A	70.5
C2—C3—H3	120.7	C19—C20—H20B	120.0
C1—C12—S2	113.3 (2)	Pd1—C20—H20B	130.4
C1—C12—H12A	108.9	H20A—C20—H20B	120.0
S2—C12—H12A	108.9	C19A—C20—H20C	120.0
C1—C12—H12B	108.9	Pd1—C20—H20C	69.0
S2—C12—H12B	108.9	C19A—C20—H20D	120.0
H12A—C12—H12B	107.7	Pd1—C20—H20D	134.3
C13—C14—C15	118.4 (5)	H20C—C20—H20D	120.0
C13—C14—H14	120.8		
			
C7—S1—C6—C5	69.5 (2)	N1—C5—C4—C3	1.1 (5)
C1—N1—C5—C4	−1.7 (4)	C6—C5—C4—C3	−176.0 (3)
Pd1—N1—C5—C4	−179.3 (2)	C13—C14—C15—C16	−0.2 (7)
C1—N1—C5—C6	175.5 (3)	C7—N2—C11—C10	1.5 (6)
Pd1—N1—C5—C6	−2.1 (3)	Pd1—N2—C11—C10	−172.4 (4)
S1—C6—C5—N1	−94.4 (3)	C14—C13—N3—C17	−1.1 (7)
S1—C6—C5—C4	82.8 (3)	S2—C13—N3—C17	176.6 (4)
C5—N1—C1—C2	0.9 (4)	C10—C9—C8—C7	−0.3 (7)
Pd1—N1—C1—C2	178.4 (2)	N2—C7—C8—C9	1.6 (6)
C5—N1—C1—C12	−179.7 (3)	S1—C7—C8—C9	−175.9 (4)
Pd1—N1—C1—C12	−2.3 (4)	C8—C9—C10—C11	−0.3 (8)
C11—N2—C7—C8	−2.2 (5)	N2—C11—C10—C9	−0.3 (7)
Pd1—N2—C7—C8	170.3 (3)	C13—N3—C17—C16	−0.9 (9)
C11—N2—C7—S1	175.0 (3)	N3—C17—C16—C15	2.3 (10)
Pd1—N2—C7—S1	−12.5 (4)	C14—C15—C16—C17	−1.7 (9)
C6—S1—C7—N2	13.8 (3)	C19A—C18—C19—C20	25.8 (6)
C6—S1—C7—C8	−168.8 (3)	Pd1—C18—C19—C20	−53.0 (5)
N1—C1—C2—C3	0.4 (5)	C19A—C18—C19—Pd1	78.8 (6)
C12—C1—C2—C3	−179.0 (3)	C19—C18—C19A—C20	−34.2 (11)
C1—C2—C3—C4	−1.0 (6)	Pd1—C18—C19A—C20	49.4 (11)
N1—C1—C12—S2	62.6 (4)	C19—C18—C19A—Pd1	−83.6 (6)
C2—C1—C12—S2	−118.1 (3)	C18—C19A—C20—C19	33.8 (12)
C13—S2—C12—C1	70.3 (3)	Pd1—C19A—C20—C19	83.2 (6)
C15—C14—C13—N3	1.7 (6)	C18—C19A—C20—Pd1	−49.5 (11)
C15—C14—C13—S2	−176.0 (3)	C18—C19—C20—C19A	−26.3 (6)
C12—S2—C13—N3	24.4 (3)	Pd1—C19—C20—C19A	−79.6 (6)
C12—S2—C13—C14	−157.8 (3)	C18—C19—C20—Pd1	53.4 (5)
C2—C3—C4—C5	0.3 (6)		

### Hydrogen-bond geometry (Å, º)

**Table:**

*D*—H···*A*	*D*—H	H···*A*	*D*···*A*	*D*—H···*A*
C3—H3···F3^i^	0.93	2.42	3.270 (8)	151
C4—H4···F2^ii^	0.93	2.47	3.358 (6)	161
C8—H8···F4^iii^	0.93	2.44	3.317 (8)	157
C12—H12*A*···F3^iv^	0.97	2.45	3.126 (8)	126

Symmetry codes: (i) −*x*+1, −*y*+1, −*z*+1; (ii) −*x*+3/2, *y*+1/2, −*z*+3/2; (iii) *x*+1/2, −*y*+1/2, *z*+1/2; (iv) *x*+1/2, −*y*+1/2, *z*−1/2.
